# Discordance diagnosis between B-mode ultrasonography and MRI proton density fat fraction for fatty liver

**DOI:** 10.1038/s41598-023-42422-5

**Published:** 2023-09-20

**Authors:** Chul-min Lee, Mimi Kim, Bo-Kyeong Kang, Dae Won Jun, Eileen L. Yoon

**Affiliations:** 1 Department of Radiology, Hanyang University College of Medicine, 222 Wangsimni-ro, Seongdong-gu, Seoul, 133-791 Korea; 2 Department of Internal Medicine, Hanyang University College of Medicine, Seoul, Korea; 3 Hanyang Institute of Bioscience and Biotechnology, Hanyang University, Seoul, Korea

**Keywords:** Gastroenterology, Medical research

## Abstract

We aimed to evaluate the frequency and causes of discordant results in fatty liver (FL) diagnosis between B-mode ultrasonography (B-USG) and magnetic resonance imaging proton density fat fraction (MRI-PDFF). We analyzed patients who underwent both B-USG and MRI-PDFF within a 6-month interval. We made a confusion matrix for FL diagnosis between B-USG and MRI-PDFF and identified four discordant groups as follows: (1) the “UFL-MnFL-wo” group [B-USG FL–MRI-PDFF no FL without chronic liver disease (CLD) or liver cirrhosis (LC)]; (2) the “UFL-MnFL-w” group (B-USG FL–MRI-PDFF no FL with CLD or LC); (3) the “UnFL-MFL-wo” group (B-USG no FL–MRI-PDFF FL without CLD or LC); and (4) the “UnFL-MFL-w” group (B-USG no FL–MRI-PDFF FL with CLD or LC). We compared the “UFL-MnFL-wo” group with the control group in terms of various parameters. We found 201 patients (201/1514, 13.3%) with discordant results for FL diagnosis between B-USG and MRI-PDFF. The “UFL-MnFL-wo” group accounted for the largest portion at 6.8% (103/1514), followed by the “UFL-MnFL-w” group (79/1514, 5.2%) and the “UnFL-MFL-w” group (16/1514, 1.1%). The mean and right PDFF values, body mass index, and abdominal wall thickness were significantly higher in the “UFL-MnFL-wo” group than in the control group (*p* ≤ 0.001). The frequency of discordant results in the diagnosis of FL between B-USG and MRI-PDFF could be identified. The causes of discordant results were that B-USG was fairly accurate in diagnosing FL disease and that accompanying CLD or LC hindered the evaluation of FL.

## Introduction

Fatty liver (FL) is a relatively common liver disease, and its prevalence has increased. An early and accurate diagnosis of FL is becoming more important because nonalcoholic fatty liver disease (NAFLD) is one of the important causes of chronic liver disease (CLD) and liver cirrhosis (LC)^1–4^. In addition, patients with NAFLD have a high risk of cardiovascular- and liver-related morbidities and mortalities, so the early diagnosis of FL is essential^5^.

Liver biopsy is the gold standard for diagnosing FL; however, it is an invasive procedure that limits its usage for all patients suspected of FL^6^. Recently, magnetic resonance imaging proton density fat fraction (MRI-PDFF) was introduced with advances in imaging techniques. MRI-PDFF can be used to accurately measure hepatic steatosis, and there have been many studies on the excellent diagnostic performance of MRI-PDFF for hepatic steatosis quantification. The hierarchical summary receiver operating characteristics of MRI-PDFF for FL diagnosis were 0.90–0.98 according to previous meta-analyses^7,8^. Furthermore, in recent studies, scholars have reported excellent linearity and reproducibility of MRI-PDFF across vendors, manufacturers, and reconstruction methods^9,10^. Therefore, MRI-PDFF is an excellent alternative to liver biopsy because of its comparable diagnostic performance^11^.

Even though there have been many advances in imaging techniques for diagnosing fatty liver, liver B-mode ultrasonography (B-USG) is still one of the most widely used imaging modalities for diagnosing FL owing to the increased supply of B-USG equipment and relatively low examination costs. A recent study with a large scale of multinational and multicenter cohorts proved the excellent diagnostic performance of B-USG for any grade of FL based on MRI-PDFF^12^. However, in daily clinical practice, B-USG and other imaging tests (including MRI-PDFF) occasionally show different results for FL diagnosis. These discordant results often lead to distrust of the diagnostic performance of B-USG. Nevertheless, B-USG remains an important screening test and a primary imaging modality for FL currently and in the future.

Therefore, we evaluated the exact frequency of discordant results for FL diagnosis between B-USG and MRI-PDFF and analyzed the causes of the discordance as an important basis for improving the diagnostic performance of B-USG for FL.

## Materials and methods

The institutional review board of Hanyang University Hospital approved this study, and the need for informed consent was waived due to its retrospective nature (IRB No. HYUH 2021-09-013). All methods were performed in accordance with the relevant guidelines and regulations.

### Study population

**Figure 1:**
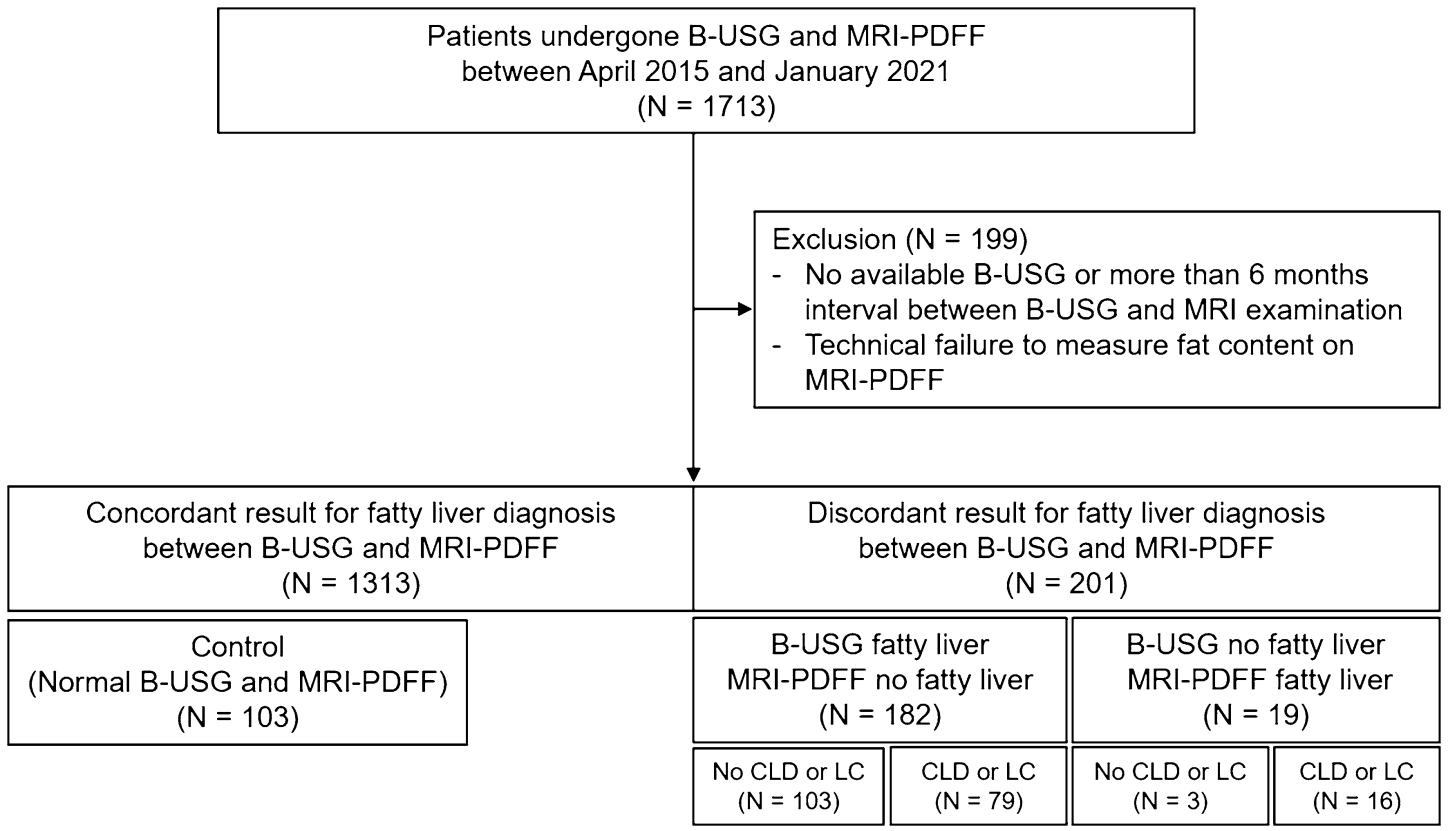
Flow chart of the study population. *B-USG* B-mode ultrasonography, *CLD* chronic liver disease, *FL* fatty liver, *LC* liver cirrhosis, *MRI-PDFF* magnetic resonance imaging proton density fat fraction.

We reviewed consecutive patients who had undergone MRI-PDFF between January 2015 and January 2021. They were referred to a tertiary referral hospital for further evaluation of liver disease of various etiologies. Among them, we included patients who had undergone B-USG within a 6-month interval of the MRI-PDFF examination date. Then, we excluded patients as follows: (1) no available B-USG or more than a 6-month interval between the B-USG and MR examination date and (2) technical failure to measure the mean PDFF value on MRI-PDFF (Fig. 1).

### B-USG examination

**Figure 2:**
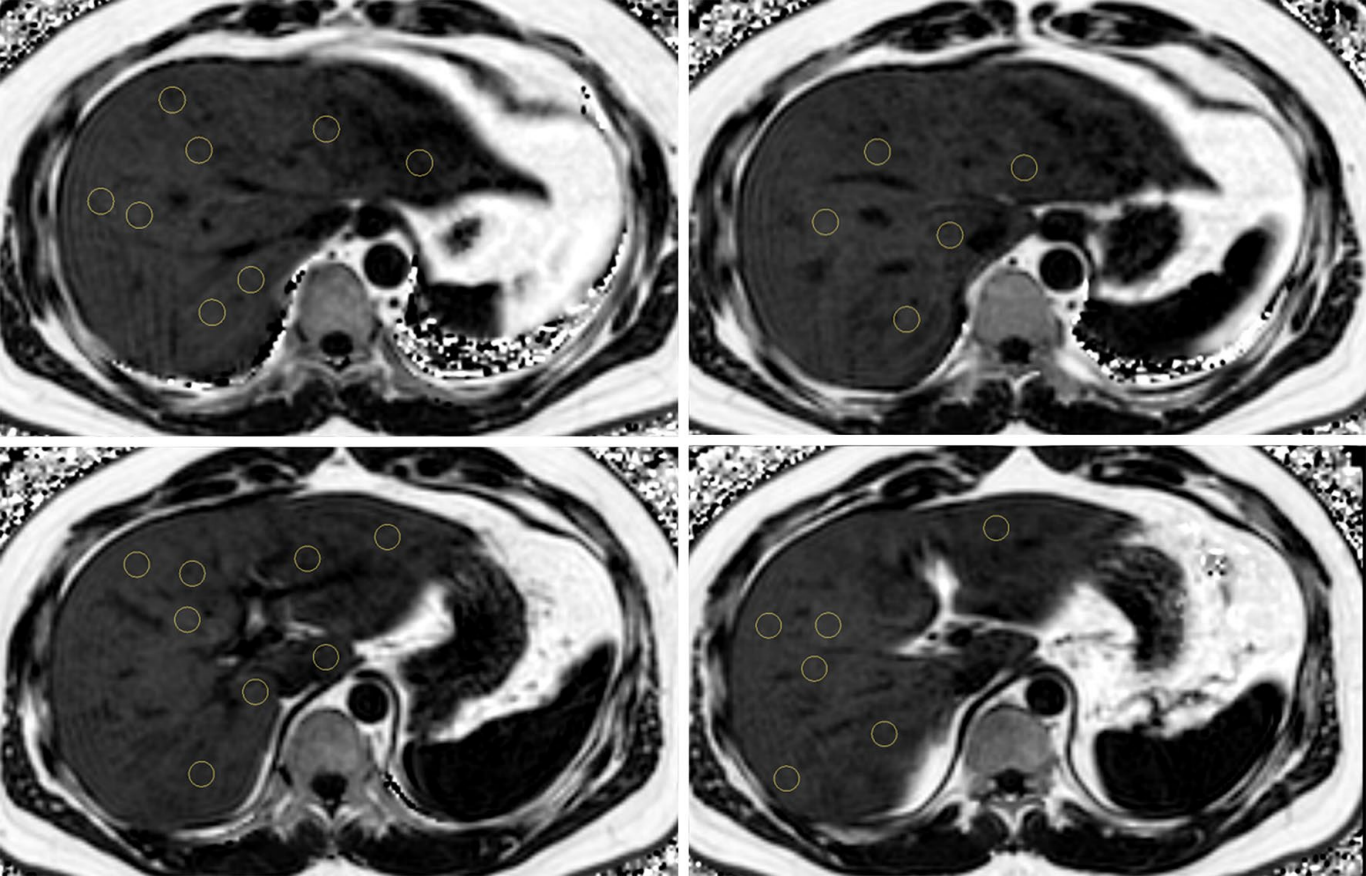
A demonstrative image of magnetic resonance imaging-proton density fat fraction (MRI-PDFF) measurement. Three circular regions of interest (100 mm^2^ area) were drawn in each hepatic segment to avoid large vessels, bile ducts, capsules, and space-occupying lesions. The average of 24 measurements in each hepatic segment was considered the mean MRI-PDFF value.

Three abdominal radiologists with 10, 6, and 4 years of experience in abdominal imaging performed standard liver scanning using four different B-USG scanners (EPIQ 5Q or IU-22, Philips Health Care, Best, Netherlands; Aixplorer, SuperSonic Imaging, Aixen-Provence, France; and RS85, Samsung Medison, Seoul, Korea). They evaluated FL disease, and FL was qualitatively graded into three stages as follows: (1) mild: mild increase in hepatic echogenicity compared to renal echogenicity; (2) moderate: increase in hepatic echogenicity compared to renal echogenicity, slightly impaired vision of the hepatic vessel wall and diaphragm; and (3) severe: marked increase in hepatic echogenicity compared to renal echogenicity, posterior shadowing, and invisible hepatic vessel wall and diaphragm. In the case of chronic renal disease or absent right kidney, in which comparison of hepatic echogenicity was difficult, we alternatively used splenic echogenicity^13,14^ (Fig. 2). Then, we retrospectively collected the data of FL diagnosis based on the B-USG report. CLD or LC was evaluated based on B-USG findings (e.g., coarseness of hepatic echogenicity or surface nodularity) and clinical presentations (e.g., results of Fibroscan™ or FIB-4).

### MRI-PDFF examination and measurement

MRI-PDFF examinations were performed using 3T MRI scanners (Ingenia or Achieva, Philips Health care, Best, the Netherlands) with a torso coil. The three-dimensional multiple gradient echo (GRE) sequence was performed in a single breath hold. The parameters of MRI-PDFF were as follows: six echo times (TE) (first TE, 0.98 ms; delta TE, 0.8 ms) and repetition time (TR), 6.3 ms; flip angle, 3°; parallel imaging SENSE factor, 2; number of signal average, 1; matrix size, 300 × 300; field-of-view, 350 × 350 mm; number of slices, 60; and a 3-mm slice thickness (50% interpolation). We used six echo acquisitions and seven peak fat models to overcome the T2* bias and fat complexity. The acquired MRI data was processed using software (ISP; Philips Health care, Best, the Netherlands). The MRI-PDFF maps, which were adjusted for confounding variables, were generated for the purpose of MRI-PDFF measurements. Then, three circular regions of interest (100 mm^2^ area) were drawn in each hepatic segment to avoid large vessels, bile ducts, capsules, and space-occupying lesions. The average of 24 measurements in each hepatic segment was considered the mean MRI-PDFF value, and the right MRI-PDFF value was calculated as the average of 12 measurements in the right hemiliver. The MRI-PDFF measurements were performed by either of two abdominal radiologists with more than six years of experience in abdominal imaging (K.M.M., K.B.K.), and we retrospectively collected the MRI-PDFF measurement data based on the MRI report. According to a previous study, we adopted a cutoff value of 6.4% (mean MRI-PDFF value ≥ 6.4%) for the presence of FL^15^. We also measured the abdominal wall thickness for 206 patients included in this study, defining it as the distance between the liver capsule and skin at the mid-axillary line on an axial T2-weighted image.

### Clinical parameters

All patients underwent laboratory tests after midnight fasting, and the following laboratory data were obtained: aspartate aminotransferase (AST), alanine transferase (ALT), triglyceride, gamma-glutamyltransferase (GGT), high-density lipoprotein (HDL), low-density lipoprotein (LDL), and cholesterol. In addition, we acquired the age, sex, and body mass index (BMI) of each patient through the electronic medical record review, and BMI was calculated using the following equation: BMI (kg/m^2^) = weight (kg)/[height (m)]^2^.

### Discordance analysis between B-USG and MRI-PDFF

**Figure 3:**
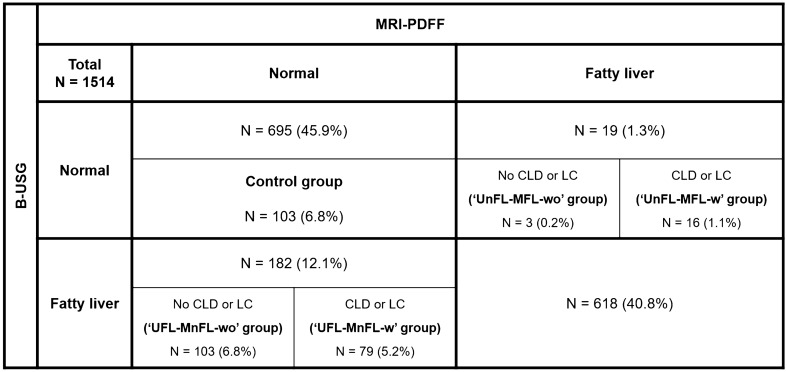
Discordant assessment of fatty liver between B-USG and MRI-PDFF. *B-USG* B-mode ultrasonography, *CLD* chronic liver disease, *LC* liver cirrhosis, *MRI-PDFF* magnetic resonance imaging proton density fat fraction.

We created a confusion matrix for FL diagnosis between B-USG and MRI-PDFF to classify concordant and discordant results between B-USG and MRI-PDFF. Ultrasonographic fatty liver (“UFL”) was defined when there was mild FL or more on B-USG. FL on MRI (“MFL”) was defined when the mean MRI-PDFF value was 6.4% or more. Patients were further categorized according to the presence or absence of CLD or LC. We identified four discordant groups as follows: (1) “UFL-MnFL-wo” group (B-USG FL–MRI-PDFF no FL without CLD or LC), (2) “UFL-MnFL-w” group (B-USG FL–MRI-PDFF no FL with CLD or LC), (3) “UnFL-MFL-wo” group (B-USG no FL–MRI-PDFF FL without CLD or LC), and (4) “UnFL-MFL-w” group (B-USG no FL–MRI-PDFF FL with CLD or LC) (Fig. 3).

We additionally included 103 patients with normal B-USG and MRI-PDFF results (i.e., no FL on B-USG and mean PDFF less than 6.4%) without CLD or LC for the comparison. We defined them as the “control group” compared to the “UFL-MnFL-wo” group. MRI-PDFF values were within the normal range (less than 6.4%) in both groups; however, B-USG showed different results for FL diagnosis in each group (no FL in the control group and FL in the “UFL-MnFL-wo” group).

### Statistical analysis

The discordance analysis yielded the exact frequency of each discordant group. We compared the “UFL-MnFL-wo” group with the control group for various imaging and clinical parameters that could cause differences between the two groups. Continuous variables were compared using the independent t test, and categorical variables were compared using the chi-squared test. We set MRI-PDFF as the reference standard for FL diagnosis instead of liver biopsy. We evaluated the diagnostic performance of B-USG for FL diagnosis in all patients and each subgroup according to the presence of CLD or LC using the area under the receiver operating characteristic curve (AUC), sensitivity, specificity, positive predictive value (PPV), and negative predictive value (NPV). Statistical significance was set at *p* < 0.05. All statistical analyses were performed using SPSS version 26.0 (IBM, Armonk, NY, USA).

### Ethics approval

Institutional Review Board of Hanyang University Hospital approved this study.

## Results

### Patient characteristics

Table 1 shows the characteristics of all 1514 patients. There were 727 women (48.0%), with a mean age of 53.2 ± 13.0 years (range 18–79 years) and a mean BMI of 25.8 ± 14.9 kg/m^2^. Among them, 368 patients (24.3%) had hepatitis B, and 54 patients had hepatitis C (3.6%). In addition, 164 patients (10.8%) had alcoholic liver disease. There were seven hundred ninety-four patients (794/1514, 52.4%) diagnosed with FL on B-USG, and the mean MRI-PDFF value was 7.9 ± 7.8%.

**Table 1:** Baseline demographics and clinical characteristics of studied patients.

	N = 1514
Age (years)	53.2 ± 13.0
Sex*	
Men	787 (52.0%)
Women	727 (48.0%)
BMI (kg/m^2^)	25.8 ± 14.9
Hepatitis B	368 (24.3%)
Hepatitis C	54 (3.6%)
Alcoholic liver disease	164 (10.8%)
Mean MRI-PDFF value (%)	7.9 ± 7.8
Right MRI-PDFF value (%)	8.7 ± 25.3
B-USG FL*	
No	720 (47.6%)
Yes	794 (52.4%)
Mild	405 (26.8%)
Moderate	319 (21.1%)
Severe	70 (4.6%)
CLD or LC*	
No	760 (50.2%)
Yes	754 (49.8%)
CLD	419 (27.7%)
LC	335 (22.1%)
Lab data	
AST (U/L)	57.2 ± 165.8
ALT (U/L)	49.9 ± 154.8
GGT (U/L)	86.5 ± 133.5
TG (mg/dL)	138.2 ± 91.5
HDL (mg/dL)	49.3 ± 17.6
LDL (mg/dL)	102.5 ± 32.1
Cholesterol (mg/dL)	178.1 ± 39.6

Data are mean ± standard deviation.

*ALT* alanine transferase, *AST* aspartate aminotransferase, *BMI* body mass index, *CLD* chronic liver disease, *GGT* gamma-glutamyltransferase, *HDL* high-density lipoprotein, *LC* liver cirrhosis, *LDL* low-density lipoprotein, *PDFF* proton density fat fraction, *TG* triglyceride, *B-USG* B-mode ultrasonography.

*Data are the number of patients (percentage).

### Discordant results for FL diagnosis between B-USG and MRI-PDFF

**Figure 4:**
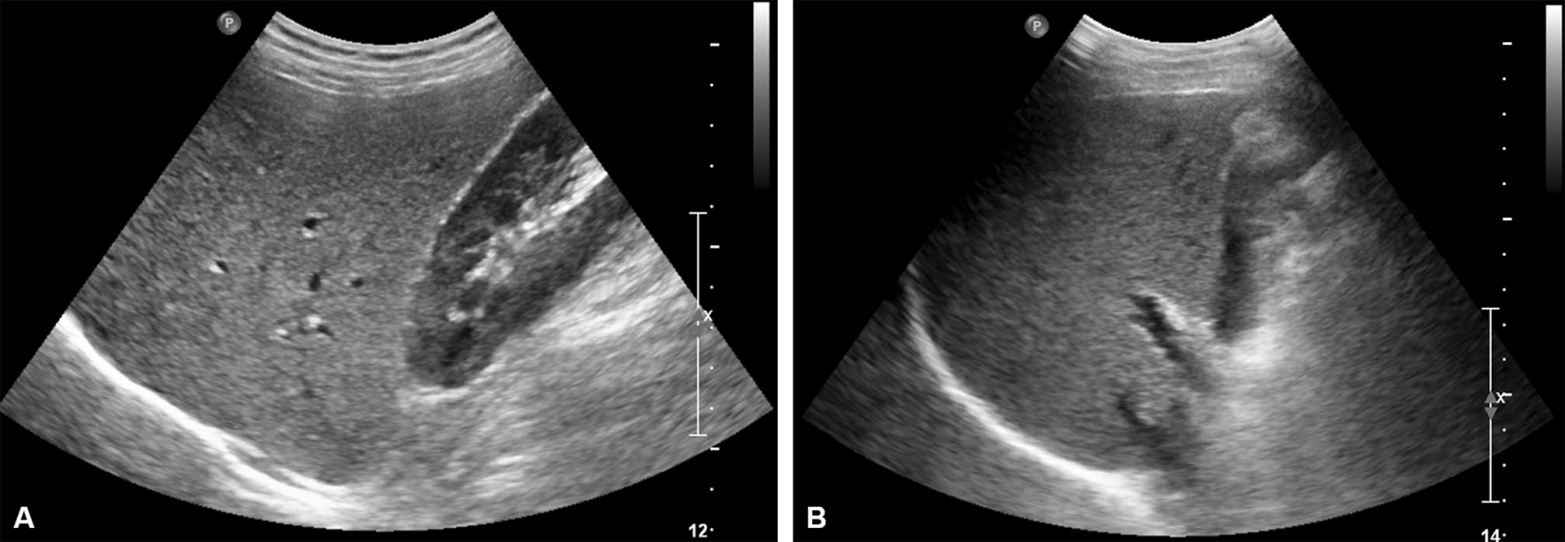
‘UFL-MnFL-wo’ group (B-USG FL–MRI-PDFF no FL without CLD or LC). A 72-year-old male with hepatitis B underwent B-USG and MRI-PDFF to evaluate fatty liver. In the B-USG (**A**,**B**), the hepatic echogenicity was increased with abnormal hepatorenal echo (**A**). In addition, the hepatic vessel walls and diaphragm were normally visualized (**B**). Features of chronic liver disease or liver cirrhosis were not observed. Then, the B-USG diagnosis was mild FL. However, the mean PDFF value was 2.9%, and the right PDFF was 3%. The patient’s BMI was 22 kg/m^2^, and the abdominal wall thickness was 1.6 cm at the mid-axillary line (not shown).

**Figure 5:**
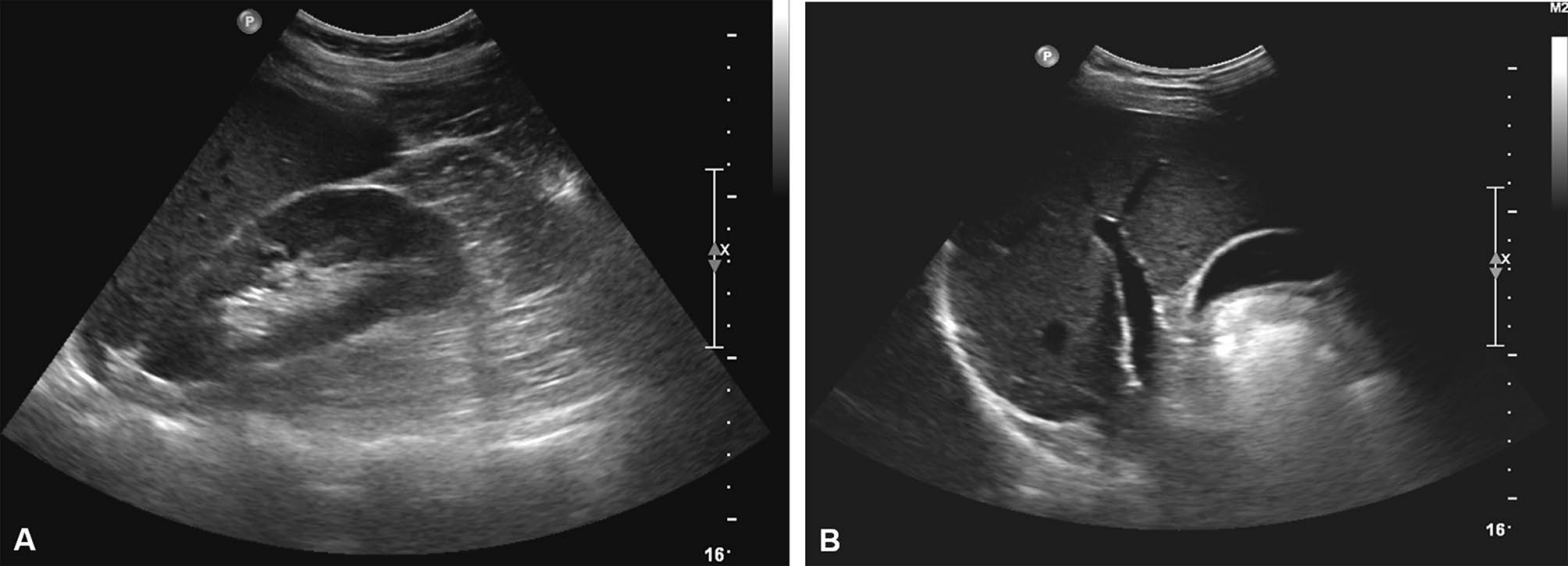
‘UnFL-MFL-wo’ group (B-USG no FL–MRI-PDFF FL without CLD or LC). A 41-year-old male patient with nonalcoholic FL disease (NAFLD) underwent B-USG and MRI-PDFF. In B-USG (**A**,**B**), the hepatic parenchymal echogenicity was similar to that of the right renal cortex (**A**). The hepatic vessel walls and diaphragm were normally visualized (**B**). Features of chronic liver disease or liver cirrhosis were not observed. The mean PDFF value was 9.1%, and the right PDFF was 11.8%. The abdominal wall thickness was 2.8 cm at the mid-axillary line (not shown).

We found 201 patients (201/1514, 13.3%) with discordant results for FL diagnosis between B-USG and MRI-PDFF. Among 201 patients with discordant results, 182 (182/1514, 12.1%) were diagnosed with FL on B-USG. However, the mean MRI-PDFF value of these patients was less than 6.4%. Among them, 103 patients (6.8%) did not have CLD or LC (“UFL-MnFL-wo” group), and 79 patients (5.2%) had CLD or LC (“UFL-MnFL-w” group). In contrast, the remaining 19 patients (1.3%) showed no FL on B-USG; however, the mean PDFF value of these patients was 6.4% or more. Sixteen patients (1.1%) had CLD or LC (“UnFL-MFL-w” group), and the remaining three patients (0.2%) had no CLD or LC (“UnFL-MFL-wo” group) (Fig. 3). In addition, representative cases are presented in Figs. 4 and 5.

### Differences between the “UFL-MnFL-wo” and control groups

The mean and right PDFF values in the “UFL-MnFL-wo” group were significantly higher than those in the control group (3.9% vs. 2.5% and 3.9% vs. 2.4%, respectively; all *p* < 0.001). Patients in the “UFL-MnFL-wo” group also had a significantly higher BMI (25.8 kg/m^2^ vs. 23.2 kg/m^2^, *p* < 0.001) and abdominal wall thickness (2.6 cm vs. 2.4 cm, *p* = 0.001) than patients in the control group. However, there were no significant differences in the laboratory data between the two groups (*p* ≥ 0.283) (Table 2).

**Table 2:** Comparison of various parameters between the ‘UnFL-MFL-wo’ group and the control group.

	‘UnFL-MFL-wo’ group ( B-USG No FL–MRI-PDFF FL without CLD or LC, N = 103)	Control group (Normal B-USG and MRI-PDFF without CLD or LC, N = 103)	*p* value
BMI (kg/m^2^)	25.8 ± 4.9	23.2 ± 3.1	< 0.001
Mean MRI-PDFF value (%)	3.9 ± 1.6	2.5 ± 1.1	< 0.001
Right MRI-PDFF value (%)	3.9 ± 1.7	2.4 ± 1.2	< 0.001
Abdominal wall thickness (cm)	2.6 ± 0.6	2.4 ± 0.5	0.001
Laboratory data			
AST (U/L)	43.4 ± 33.8	46.1 ± 37.4	0.589
ALT (U/L)	45.4 ± 54.1	45.2 ± 69.8	0.973
GGT (U/L)	90.9 ± 149.7	76.5 ± 97.4	0.458
TG (mg/dL)	131.0 ± 69.8	132.1 ± 111.5	0.935
HDL (mg/dL)	52.1 ± 18.0	53.7 ± 17.9	0.544
LDL (mg/dL)	107.6 ± 36.7	102.8 ± 31.4	0.350
Cholesterol (mg/dL)	182.4 ± 39.2	176.4 ± 39.9	0.283

Data are mean ± standard deviation.

*ALT* alanine transferase, *AST* aspartate aminotransferase, *BMI* body mass index, *CLD* chronic liver disease, *FL* fatty liver, *GGT* gamma-glutamyltransferase, *HDL* high-density lipoprotein, *LC* liver cirrhosis, *LDL* low-density lipoprotein, *PDFF* proton density fat fraction, *TG* triglyceride, *B-USG* ultrasonography.

### Diagnostic performance of B-USG for diagnosing FL

The agreement between B-USG and MRI-PDFF was 0.694 (*p* < 0.001), with a sensitivity of 95.8% [95% confidence interval (CI) 93.8–97.2%], specificity of 76.9% (95% CI 74.1–79.7%), and accuracy of 84.5% (95% CI 82.9–87.1). Within a subgroup of 760 patients without CLD or LC, the sensitivity and specificity were 98.2% (95% CI 96.5–99.2) and 59.4% (95% CI 53.7–64.8), respectively. The sensitivity was 89.2% (95% CI 83.5–93.5), and the specificity was 86.4% (95% CI 83.3–89.0) in the remaining 754 patients with CLD or LC (Table 3).

**Table 3:** Diagnostic performance of B-mode ultrasonography for diagnosing fatty liver.

	AUC (95% CI)	Sensitivity (95% CI)	Specificity (95% CI)	PPV (95% CI)	NPV (95% CI)
All patients (N = 1514)	0.863 (0.845–0.880)	95.8% (93.8–97.2)	76.9% (74.1–79.7)	73.8% (71.4–76.1)	96.4% (94.8–97.5)
Patients without CLD or LC (N = 760)	0.788 (0.757–0.816)	98.2% (96.5–99.2)	59.4% (53.7–64.8)	77.3% (74.9–79.6)	95.9% (92.1–97.9)
Patients with CLD or LC (N = 754)	0.878 (0.852–0.900)	89.2% (83.5–93.5)	86.4% (83.3–89.0)	65.1% (60.1–69.7)	96.6% (94.8–97.8)

*AUC* the area under the receiver operating characteristic curve, *CI* confidence interval, *CLD* chronic liver disease, *LC* liver cirrhosis, *NPV* negative predictive value, *PPV* positive predictive value.

## Discussion

Our study showed the frequency of discordant results for FL diagnosis between B-USG and MRI-PDFF. Out of 1514 patients, 201 patients (201/1514, 13.3%) showed discordant results between B-USG and MRI-PDFF. The “UFL-MnFL-wo” group accounted for the largest proportion at 6.8% (103/1514), followed by the “UFL-MnFL-w” group (79/1514, 5.2%) and the “UnFL-MFL-w” group (16/1514, 1.1%). The “UnFL-MFL-wo” group showed the lowest proportion at 0.2% (3/1514).

We focused on the “UFL-MnFL-wo” group, which showed the largest proportion of discordant results among the four discordant groups. We compared the “UFL-MnFL-wo” group with the control group because both groups showed a normal range of mean PDFF values (less than 6.4%); however, FL was successfully identified using B-USG in the “UFL-MnFL-wo” group and not in the control group. Although the mean PDFF values of the “UFL-MnFL-wo” and control groups were lower than the cutoff of 6.4% for FL diagnosis, the mean PDFF values of the “UFL-MnFL-wo” group were higher than those of the control group. This result could mean that B-USG could accurately discriminate the differences in the mean PDFF values between the two groups. In addition, the BMI of the “UFL-MnFL-wo” group was higher than that of the control group. In general, patients with higher BMI might have higher hepatic fat content than those with lower BMI, which was reflected by the higher mean PDFF value in the “UFL-MnFL-wo” group than in the control group^16^.

We measured and compared abdominal wall thicknesses in all 206 studied patients because we assumed that abdominal wall thickness could lead to overestimation or underestimation of hepatic echogenicity in comparing renal echogenicity. In our study, the abdominal wall thickness was higher in the “UFL-MnFL-wo” group than in the control group, which indicates that patients with thicker abdominal walls (“UFL-MnFL-wo” group) showed increased hepatic echogenicity compared with those with thinner abdominal walls (control group). However, the difference in the mean abdominal wall thickness was small (0.2 cm). These small differences might not affect hepatic echogenicity in B-USG images in routine clinical practice. Otherwise, our results might be due to the characteristics of our patient group (Asian population), which included a small number of obese patients. There might have been different results in a group containing many obese patients with thick abdominal walls, such as in the Western population.

Several previous studies, especially for NAFLD, have shown that hepatic echogenicity is likely to increase in patients with elevated liver enzymes^17–19^. Because the “UFL-MnFL-wo” group had a higher mean PDFF value than the control group in our study, the “UFL-MnFL-wo” group might be expected to show a higher level of liver enzymes than the control group. However, the two groups showed no significant differences in any laboratory parameters. These results may be because the difference in the mean PDFF values between the two groups was insufficient to cause pathophysiological abnormalities in the liver.

In our study, three patients (0.2%) with a mean PDFF value of 6.4% or more were not diagnosed with FL in the B-USG (“UnFL-MFL-wo”) group. The low false-negative results of our study differed from those of previous studies, which yielded low sensitivity of B-USG for FL (range of sensitivity 49.8–66.6%) because of many false-negative results^1,20,21^. Until now, B-USG has played an important role as a screening test in diagnosing FL; however, its weakness has been the high false-negative rates. However, the B-USG sensitivity for FL diagnosis in our study was high at 95.8%, which might be attributed to the low interreader variability (only three readers) and a high interest in FL diagnosis shown by the B-USG examiners in our institution.

According to a previous study, we set the cutoff of 6.4% for FL diagnosis in the MRI-PDFF^15^. However, many studies have reported various cutoff values for FL diagnosis in MRI-PDFF; 5% and 6.4% are the most commonly used. When we changed the cutoff value from 6.4 to 5%, the sensitivity changed from 95.8 to 92.4%, which was still higher than that in previous studies^1,20,21^.

Among patients with discordant results between B-USG and MRI-PDFF, the “UFL-MnFL-wo” and “UFL-MnFL-w” groups accounted for the second- and third-largest proportions, respectively. A coarse and increased echotexture of the liver is a well-known B-USG finding in CLD and LC^22^. Therefore, the altered hepatic echogenicity due to CLD or LC might be interpreted as FL by the B-USG examiner. In contrast, the examiner might interpret the increased hepatic echogenicity as the change by CLD or LC, missing the presence of FL. Therefore, more attention is recommended when diagnosing FL in patients with CLD or LC.

We did not use B-USG quantification techniques developed using several types of equipment because they were not yet practically applicable in the actual ultrasonography examination^23^.

Our study has some limitations. First, our study was limited by its retrospective nature and the small number of patients. Second, MRI-PDFF was used as the reference standard for FL diagnosis instead of liver biopsy. Although liver biopsy is the gold standard for FL diagnosis, it is invasive, and MRI-PDFF is regarded as an excellent alternative to liver biopsy because of its superior diagnostic performance. Finally, we set the interval between B-USG and MRI-PDFF to 6 months or less. The duration of 6 months encompasses a sufficiently extended temporal span during which alterations in the extent or manifestation of steatosis may have transpired owing to modifications in lifestyle, the discontinuation of alcohol consumption or the implementation of antiviral therapeutic regimens. However, it is noteworthy that upward of 90% of the participants enrolled in the study underwent MRI-PDFF examinations within a span of 3 months after undergoing ultrasonography via B-USG.

In conclusion, we determined the frequency of discordant results for FL diagnosis between B-USG and MRI-PDFF. The causes of the discordances were that B-USG was fairly accurate in diagnosing FL disease, discriminating the differences in mean PDFF below the cutoff value. In addition, the accompanying CLD or LC might hinder the evaluation of FL because of the alteration in hepatic echogenicity. Hence, drawing upon the findings of our study, there is the potential to solidify the role of B-USG, a widely utilized primary imaging modality for diagnosing fatty liver, while enhancing confidence in precision. Moreover, considering the impediment posed by CLD or LC to accurate fatty liver diagnosis, B-USG operators should approach the examination cautiously. The results of this study could be reinforced through the implementation of subsequent follow-up studies within the patient cohort in primary care settings, encompassing a larger number of patients.

## Data Availability

The datasets generated and/or analyzed during the current study are not publicly available due to personal information protection but are available from the corresponding author on reasonable request.

## Abbreviations

AUC: Area under the receiver operating characteristic curve
B-USG: B-mode ultrasonography
CLD: Chronic liver disease
FL: Fatty liver
LC: Liver cirrhosis
MRI-PDFF: Magnetic resonance imaging proton density fat fraction
NAFLD: Nonalcoholic fatty liver disease
NPV: Negative predictive value
PPV: Positive predictive value
UFL-MnFL-wo: B-USG FL–MRI-PDFF no FL without CLD or LC
UFL-MnFL-w: B-USG FL–MRI-PDFF no FL with CLD or LC
UnFL-MFL-wo: B-USG no FL–MRI-PDFF FL without CLD or LC
UnFL-MFL-w: B-USG no FL–MRI-PDFF FL with CLD or LC
